# Enzyme Hydrolysates from *Stichopus horrens* as a New Source for Angiotensin-Converting Enzyme Inhibitory Peptides

**DOI:** 10.1155/2012/236384

**Published:** 2012-08-15

**Authors:** Bita Forghani, Afshin Ebrahimpour, Jamilah Bakar, Azizah Abdul Hamid, Zaiton Hassan, Nazamid Saari

**Affiliations:** ^1^Department of Food Science, Faculty of Food Science and Technology, Universiti Putra Malaysia, 43400 Serdang, Malaysia; ^2^Department of Food Technology, Faculty of Food Science and Technology, Universiti Putra Malaysia, 43400 Serdang, Malaysia; ^3^Faculty of Science and Technology, Universiti Sains Islam Malaysia (USIM), 71800 Bandar Baru Nilai, Malaysia

## Abstract

*Stichopus horrens* flesh was explored as a potential source for generating peptides with angiotensin-converting enzyme (ACE) inhibitory capacity using 6 proteases, namely alcalase, flavourzyme, trypsin, papain, bromelain, and protamex. Degree of hydrolysis (DH) and peptide profiling (SDS-PAGE) of *Stichopus horrens* hydrolysates (SHHs) was also assessed. Alcalase hydrolysate showed the highest DH value (39.8%) followed by flavourzyme hydrolysate (32.7%). Overall, alcalase hydrolysate exhibited the highest ACE inhibitory activity (IC_50_ value of 0.41 mg/mL) followed by flavourzyme hydrolysate (IC_50_ value of 2.24 mg/mL), trypsin hydrolysate (IC_50_ value of 2.28 mg/mL), papain hydrolysate (IC_50_ value of 2.48 mg/mL), bromelain hydrolysate (IC_50_ value of 4.21 mg/mL), and protamex hydrolysate (IC_50_ value of 6.38 mg/mL). The SDS-PAGE results showed that alcalase hydrolysate represented a unique pattern compared to others, which yielded potent ACE inhibitory peptides with molecular weight distribution lower than 20 kDa. The evaluation of the relationship between DH and IC_50_ values of alcalase and flavourzyme hydrolysates revealed that the trend between those parameters was related to the type of the protease used. We concluded that the tested SHHs would be used as a potential source of functional ACE inhibitory peptides for physiological benefits.

## 1. Introduction

Hypertension, the leading cause of cardiovascular disease in the world, is one of the most common disease affecting humans [1]. It is an important public health challenge due to its strong association with increasing death rate and consequently impacting high medical cost to the society. With the globally growing incident of hypertension, scientists have been striking to explore various foodstuffs from the functionality perspective, as potential sources of natural bioactive compounds with physiological benefits that can reduce the risk of chronic diseases. Among these compounds, are bioactive peptides exhibiting inhibitory effect on angiotensin conversing-enzyme. 

Angiotensin-converting enzyme (ACE) which is responsible for the elevation of blood pressure acts as an exopeptidase that converts an inactive form of decapeptide (angiotensin-I) to a potent vasoconstrictor, an octapeptide (angiotensin-II), and inactivates the catalytic function of bradykinin, exhibiting depressor action [1, 2]. Peptides with specific amino acid pattern at N- and C-terminal are able to inhibit ACE. Such peptides which are intact within the original protein can only exhibit their inhibition upon releasing by proteolytic enzymes. 

First peptides that showed such ACE inhibitory activity were isolated from snake venom [3]. Since then, many sources have been explored for the production of these peptides either through enzymatic hydrolysis or microbial fermentation such as tilapia [4] and chickpea [5], bovine gelatine [6], sea bream scales [7], yellowfin sole frame protein [8], oyster proteins [9], shark meat [10], and *Acaudina molpadioidea* gelatine hydrolysate [11]. 

Sea cucumber is one of those sources that has not yet been fully evaluated. It is known as the ginseng of the sea and in Asian countries there is a strong belief on its medicinal properties such as nourishing blood, tonifying kidney, moistening dryness of the intestines, wound healing, treatment of stomach ulcers, asthma, hypertension, rheumatism, and sinus [12, 13]. Sea cucumbers are flexible, elongated echinoderms, belonging to the class of Holothuroidea, the animals of which typically reside on the sea floor. The diet of most sea cucumbers consists of plankton and decaying material found in the sea. There exist over 1,100 species of sea cucumbers distributed around the world. Sea cucumbers, informally named as *trepang*, *beche-de-mer*, or *gamat*, are in use for human consumption [13]. The strong belief of health promoting effects has lead to traditional process of sea cucumber. The whole sea cucumber is anaerobically fermented for up to one year before being used as a traditional medicine. The fermented *gamat* is commercially liquid as liquid *gamat*. However, there are possibilities of making such fermentation process shorten by using proteases. 

A number of studies have already evaluated some aspects of sea cucumber healing properties like, contribution of branched fatty acids of **Stichopus chloronotus ** to potential health benefits, especially in wound healing [2], inhibition of PC3 cells proliferation in prostate malignancy in humans [14], biological functions of triterpens glycoside isolated from sea cucumber such as antifungal, cytotoxic, hemolytic, cytostatic and immunomodulatory activities [15, 16]. However, other properties such as antihypertension have not yet been supported by much scientific data. 

Therefore, in perspectives of the medicinal attributes of sea cucumber, a large potential exists to produce and evaluate sea cucumber enzyme hydrolysates as a viable source of ACE inhibitory peptides for decreasing blood pressure in hypertensive individuals. The present study explores enzymatic hydrolysis of sea cucumber (*S. horrens*) with the main objective to evaluate ACE inhibitory activity of the hydrolysates produced. 

## 2. Materials

*S. horrens* was obtained from Langkawi Island, Kedah, Malaysia. Alcalase 2.4L, protamex, and flavourzyme were purchased from Novozymes A/S (Bagsvaerd, Denmark). Trypsin, HHL (Hip-His-Leu), and ACE (derived from rabbit lung) were obtained from Sigma Chemical Co. (St. Louis, MO, USA). Papain and bromelain were purchased from Merck (Darmstadt, Germany). All other reagents used in this study were of analytical grade. 

## 3. Methods 

### 3.1. Proximate Composition

Proximate composition (moisture, ash, protein and fat contents) of sea cucumber flesh was investigated according to the AOAC methods [17]. Total nitrogen content of sea cucumber determined by Kjeldahl method was used to calculate crude protein by multiplying it with a conversion factor of 6.25 [17]. 

### 3.2. Proteolysis of *S. horrens*

Sea cucumber flesh (80 g) was homogenized with 160 mL distilled water in a Waring blender for 1 min. The enzyme concentration used was 2103 U/mL [18]. The pH and temperature of the mixture were adjusted to the optimum condition for each enzyme (alcalase, pH 7.5, 55°C; bromelain, pH 7, 45°C; flavourzyme, pH 6.5, 52.5°C; trypsin, pH 8, 37°C; papain, pH 8, 55°C; protamex, pH 7.5, 50°C) and the hydrolysis was conducted for 5 h. During the hydrolysis, the pH of the mixture was maintained at the desired level by adding 1 N NaOH or 0.5 N HCl. After 5 h hydrolysis, the process was terminated by raising the temperature of the reaction mixture to 95°C, for 10 min, to inactivate the enzyme followed by cooling to room temperature. The sea cucumber hydrolysate was then centrifuged at 10,000 ×g for 20 min to separate insoluble and soluble fractions. Finally, the soluble phase recovered was freeze-dried and preserved at −20°C, until used for further analyses. 

### 3.3. Characteristics of *S. horrens* Enzyme Hydrolysates 

#### 3.3.1. Determination of Angiotensin I-Converting Enzyme Inhibition Activity

Angiotensin I-converting enzyme (ACE) inhibition assay was performed following the method as described by Cushman and Cheung (1971) [19] with slight modifications. A 50-*μ*L volume, containing varying concentration of sea cucumber enzyme hydrolysates, was preincubated with 20 *μ*L (100 mU/mL) ACE solution at 37°C for 10 min. The mixture was then incubated with 100 *μ*L substrate containing 12.5 mM hippuryl-L-histidyl-L-leucine (HHL), 100 mM borate buffer (pH 8.3), and 300 mM NaCl at 37°C for 60 min followed by the termination of the reaction by the addition of 125 *μ*L of HCl. The released hippuric acid (HA) was extracted with 850 *μ*L ethyl acetate. After centrifugation at 4000 ×g, for 5 min, 650 *μ*L of ethyl acetate was added into a test tube and excess solvent was evaporated at room temperature under vacuum for 2 h. The HA was dissolved in 1 mL of distilled water, and the absorbance was recorded at 228 nm using a spectrophotometer (U-2800, Hitachi, Tokyo, Japan). The average value for three determinations at each concentration was used to calculate ACE inhibition rate as represented by the following equation:

(1) $$\begin{array}{c} \text{ACE}\;\text{inhibition}\;\left(\%\right)=\left[\frac{B-A}{B-C}\right]\times 100, \end{array}$$

where *A* is the absorbance of HA produced in the presence of ACE, substrate and ACE inhibitor component; *B* is the absorbance of HA produced in the presence of ACE and substrate without ACE inhibitor component, and *C* is the absorbance of HA produced in the presence of substrate without ACE and ACE inhibitor component. IC_50_ value was defined as the concentration of hydrolysate (mg/mL) which inhibited ACE activity by 50%. 

#### 3.3.2. Amino Acid Composition

Amino acid composition was studied using a reversed-phase high-performance liquid chromatography (HPLC) system [20]. A Waters HPLC (Hitachi Instruments, Tokyo, Japan) system was used. Freeze dried sea cucumber enzyme hydrolysates were used for amino acid analysis. The samples were hydrolyzed with 6 N HCl for 24 h at 110°C and then derivatized by phenylisothiocyanate. A 20-*μ*L of the derivatized sample was injected into the HPLC system equipped with photodiode array detector (model MD-2010; JASCO, Tokyo, Japan). A C_18_-reversed phase column (Thermal C_18_ 5U, 250 × 4.6 mm) maintained at 43°C was used for separation purposes. The mobile phase consisting of buffer A (0.1 M ammonium acetate, pH 6.5) and buffer B (0.1 M ammonium acetate containing acetonitrile, methanol, 44 : 46 : 10, v/v, pH 6.5) was flushed through the column at a flow rate of 1 mL/min using a linear gradient system. The UV absorption detector at a wavelength of 254 nm was employed to monitor amino acids. The identification of unknown amino acids was based on the comparison of their retention times with those of pure standards, whereas for quantification purposes standard calibration curves were used. The results were analyzed and computed by the Borwin chromatography software (Version 1.5, Jasco Co. Ltd., Japan).

#### 3.3.3. Sodium Dodecyl Sulfate Polyacrylamide Gel Electrophoresis (SDS-PAGE)

SDS-PAGE was performed according to the method of Laemmli [21]. Briefly, *S. horrens* hydrolysate samples were mixed 1 : 1(v/v) with the loading dye. The stacking gel concentration was 4% and resolving gel 15%. Gel was run at 30 mA. Protein bands were stained by silver nitrate kit (Sigma Aldrich).

#### 3.3.4. Protein Concentration

Protein concentration was determined following the method of Bradford using micro protein kit from Sigma Aldrich.

#### 3.3.5. Determination of the Degree of Hydrolysis

The degree of hydrolysis (DH) is defined as the percent ratio of the number of peptides dissociated (*h*) to the total number of peptide bonds in the substrate studied (*h*_tot_). In each case, calculation was based on the amount of NaOH or HCl added to maintain the pH constant during the hydrolysis using the following equation [18]:

(2) $$\begin{array}{c} \text{DH}\;\left(\%\right)=\frac{h}{h_{\text{tot}}}\times 100=\frac{B\times \text{Nb}}{\text{MP}}\times \frac{1}{\alpha }\times \frac{1}{h_{\text{tot}}}\times 100, \end{array}$$

where *B* is the amount of acid or base consumed (mL) to keep the pH constant during the hydrolysis. Nb is the normality of acid or base, MP is the mass (g) of protein (*N* × 6.25), and *α* is the average degree of dissociation of the *α*-NH_2_ groups released during hydrolysis expressed as

(3) $$\begin{array}{c} \alpha =\frac{10^{\text{pH}-pK}}{1+10^{\text{pH}-pK}}, \end{array}$$

where pH is the value at which the hydrolysis was conducted. The *pK* values were calculated using the equation below [22]

(4) $$\begin{array}{c} pK=7.8+\frac{298-T}{298\;T}\times 2400, \end{array}$$

where, *T* is the hydrolysis temperature in Kelvin. 

The total number of peptide bonds (*h*_tot_) in sea cucumber hydrolysate was calculated 3.44 meq/g. 

#### 3.3.6. Statistical Analysis

All experiments were performed in triplicate. Statistical significant differences among means of experimental design was studied by analysis of variance (ANOVA) at *p* ≤ 0.05 using MINITAB RELEASE 14. The values were reported as mean ± SD for triplicate determinations.

## 4. Results and Discussion

### 4.1. Proximate Composition of Sea Cucumber

The protein content of *S. horrens* as the substrate of the proteolysis was measured. The result of proximate composition of *S. horrens* for protein, moisture, fat and ash contents was as 2.83, 93.10, 0.21 and 2.70 g/100 g (fresh weight), respectively. The protein content (2.83 g/100 g) determined in this study was lower than that reported for other species of sea cucumber namely *Cucumaria frondosa *and *Holothuria tubulosa *[23]. Meanwhile, its high moisture content is not unexpected because most sea foods contain high level of moisture [21, 23]. The ash and fat contents determined in the present experiment were similar to those reported for *Parastichopus parvimensise*, *Parastichopus californicus* [24], *Acaudina molpadioides* [25], *Holothuria tubulos* [23], and *Apostichopus japonicus* [26]. 

### 4.2. Enzymatic Proteolysis of Sea Cucumber

**Figure 1:**
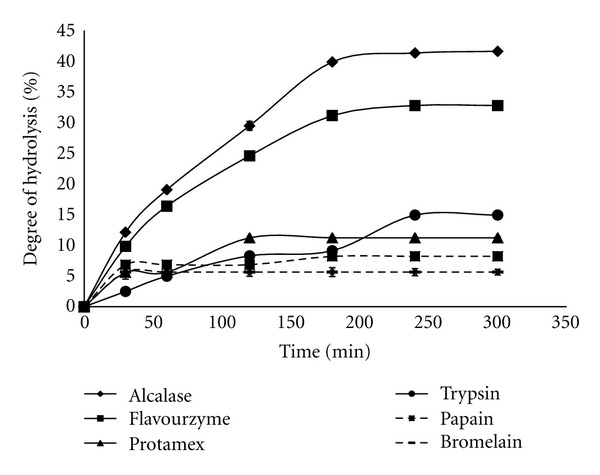
Effect of hydrolysis time on the degree of hydrolysis of *S. horrens* hydrolysates using different enzymes. Results are reported as mean ± SD. Error bar denotes standard deviation.

In order to produce ACE inhibitory peptides, sea cucumber flesh was hydrolysed with 6 different proteases. Proteases degrade protein and produce peptides with various lengths and sequences. Degree of hydrolysis (DH) is therefore an indicator of such hydrolysis progress which reflects the ability of proteases to access available cutting sites of the protein. DH, thereby, is defined as the proportion of peptides bond cleaved during proteolysis to the total peptide bond available in the protein structure. DH, in the present study, was determined for two purposes: (a) to find out the trend of DH values over the hydrolysis time; (b) to have an idea wheather the DH changes during time course can reflect the variation in IC_50_ value or not. Hence, the DH values of sea cucumber hydrolysates were analyzed and presented in Figure 1. Two distinct hydrolysis stages can be recognized during the enzymatic hydrolysis. During the first stage, the DH value steadily increased while the second stage initiated when the DH a steady phase. The first stage of hydrolysis reflects the number of cutting sites available to each proteolytic enzyme; while the steady phase is a result of either a limitation on the available cutting sites, enzyme denaturation and/or product inhibition. Among the different enzymes, alcalase hydrolysis exhibited the most rapid first phase and effective hydrolysis with the highest DH value of 39.8% compared to other proteases (Figure 1). The rapid stage of hydrolysis for alcalase as well as flavourzyme continued up to 180 min where the DH for alcalase was significantly (*P* ≤ 0.05) higher than flavourzyme.

The presently recorded asymptotic curves of alcalase and flavourzyme are similar to other studies using flavourzyme and crude extract enzymes from *Bacillus licheniformis* NH1 reported for seafood protein like, sardine [27], pacific whiting solid waste [28], sardinelle by-product [28], threadfin bream [29], *Salmo salar* [30], cuttlefish by-products [31], and cuttlefish muscle protein [32]. However, the DH values obtained with trypsin, protamex, papain, and bromelain did not exceed the level of 14.9, 11.2, 8.2, 5.6%, respectively, during 300 min of incubation. All the proteolysis curves showed that the flesh of *S. horrens* is degradable by all six proteases, and the differences among their DH values imply that the number of available cutting sites for alcalase is much higher than the others. 

### 4.3. IC_50_ of *S. horrens* Hydrolysate over the Hydrolysis Time

**Figure 2:**
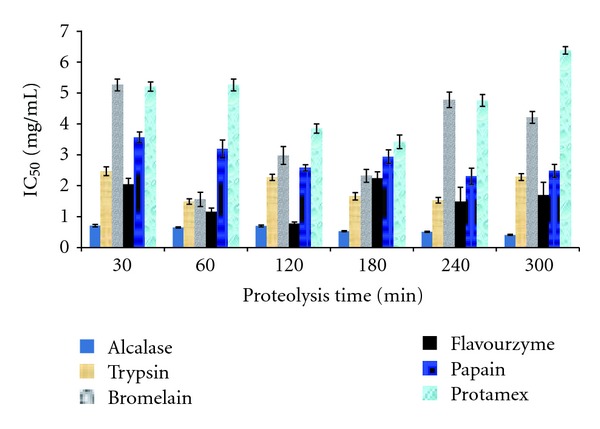
ACE inhibitory activity (IC_50_ mg/mL) of *S. horrens* hydrolysates as affected by proteolysis time. Error bar denotes standard deviation.

In order to study the change of IC_50_ over time, *S. horrens* was hydrolysed using different proteases for up to 300 min (Figure 2). Hydrolysed samples collected at 30, 60, 120, 180, 240, and 300 min were analysed for IC_50_ determination. The hydrolysates produced with all six enzymes showed considerable ACE inhibitory activity varying over a wide range of IC_50_ from 0.41 to 6.38 mg/mL. Among the tested sea cucumber hydrolysates, alcalase hydrolysate exhibited minimum IC_50_ value ranging from 0.71 mg/mL (at 30 min of hydrolysis) to 0.41 mg/mL (at 300 min of hydrolysis), indicating highest ACE inhibitory activity. However, IC_50_ for the other enzyme hydrolysates over the time course of 300 min hydrolysis varied from 2.47 to 1.49 mg/mL (trypsin), 5.26 to 1.56 mg/mL (bromelain), 2.24 to 0.78 mg/mL (flavourzyme), 3.57 to 2.31 mg/mL (papain), and 6.38 to 3.42 mg/mL (promatex). An overall decrease in IC_50_ value with the increase of hydrolysis time reflects that hydrolysis is effective towards enhancing ACE inhibitory capacity of the hydrolysates. 

Variation in IC_50_ over time can be explained from two aspects: changes in the number of amino acids in peptide chain and most importantly the amino acid residues presented as N- or C-terminal sequence of peptide. Small peptides with certain amino acids in their N- and C-terminal sequences are shown to exhibit high ACE inhibitory effect [31]. It was demonstrated that peptides containing hydrophobic (aromatic or branched side chains) amino acid residues at each of the three positions in C-terminal possess high inhibitory effect [33, 34]. The occurrence of Arg and Lys on C-terminal also contributes substantially to the inhibitory activity [33, 35]. Generation of small peptides over time depends on the capability of enzyme to degrade the protein while an N- and C-terminal sequence of a peptide is related to enzyme specificity. Therefore, changes in IC_50_ values of hydrolysates could have resulted from the changes occurred in the two terminals of generated peptides, the number of amino acids and their proportions in the crude hydrolysates at any time. Alcalase degrades peptide bond of aliphatic or aromatic amino acids such as Leu, Phe, Tyr and Trp [36]. While trypsin hydrolyzes only peptide bond with Arg or Lys and produces peptides with Arg or Lys at C-terminal [37].

Several researchers have studied the applications of different proteases on various sources to investigate their ability for producing ACE inhibitory peptides [38, 39]. However, the comparison of the present data with the literature is rather difficult as the hydrolysis conditions and choice of enzyme and enzyme concentration used were not similar. However, the present values of IC_50_ were lower than those reported for some other marine hydrolysates like oyster, scallop, codfish skin, and herring skin (>10 mg/mL) [38] but higher than the value for bonito (0.029 mg/mL [40]. The IC_50_ values of whole bovine plasma hydrolysates produced by alcalase and papain (2.53 and 17.19 mg/mL, resp.) [38] were much higher than those obtained in the present study due to the differences in their original protein sequence. On the other hand, IC_50_ values of albumin hydrolyzed with alcalase and casein degraded by trypsin was quite close to that determined presently for alcalase hydrolysate of *S. horrens*. IC_50_ value of 0.615 mg/mL for the fraction containing peptides of molecular weight lower than 2 kDa from *Acaudina molpadiodea *has been reported [11]. Likewise, during the hydrolysis of *Brachionus rotundiformis*, using different proteases (i.e., alcalase, trypsin, and papain), alcalase produced a mixture of peptides exhibiting IC_50_ value of 0.63 mg/mL [41]. Similarly in another study, the gastrointestinal digest of *Bombyx mori* showed an IC_50_ value of 0.697 mg/mL which was close to the one from Alaska Pollack skin hydrolysate [39, 42], while IC_50_ value of alcalase hydrolysate of *Spirulina platensis *(a blue-green filamentous alga) was 0.47 mg/mL [43]. These results are quite close to the values determined for alcalase sea cucumber hydrolysate in this study. Therefore, such variation observed in the inhibitory effect of abovementioned sources and different proteolysis (in this study) might be attributed to the primary and 3D structure of protein and ultimately the enzyme specificity which may have led to the cleavage of different peptide bonds and production of peptides with varying N- and C-terminal sequences affecting the ACE inhibitory activity. Thus, it can be concluded that alcalase specificity is much more compatible to the available cutting sites of *S. horrens* flesh and that resulting mixture of peptides was more efficient towards inhibition of ACE.

### 4.4. Relationship between IC_50_ and DH

**Figure 3:**
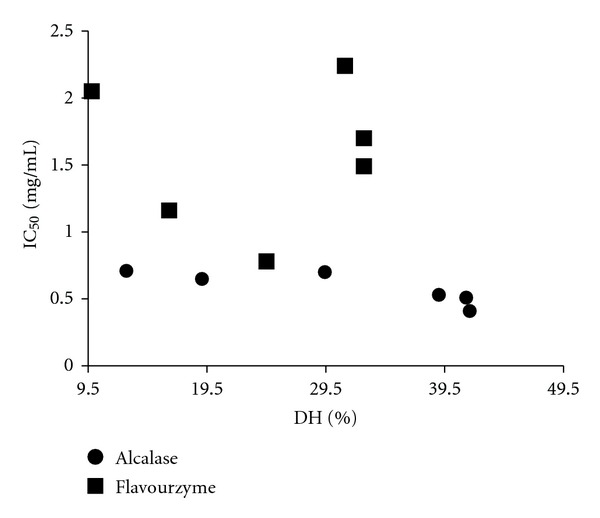
Relationship between DH and IC_50_ value of *S. horrens* hydrolysed by alcalase and flavourzyme.

DH indicates the progress of proteolysis and generation of small peptides while IC_50_ values depend on both size and N- and/or C-terminal sequences of the peptide. Therefore, it is crucial to investigate any relationship that might exist between DH and IC_50_ (Figure 3). Both hydrolysates generated by alcalase and flavourzyme represented high DH values; however, they considerably varied in IC_50_ values. Although alcalase and flavourzyme hydrolysate representing high DH value of 39.8% and 32.7% at 300** **min, respectively, yet a distinctive difference in their IC_50_ values (0.41 and 1.7** **mg/mL) exists. Plotting DH and IC_50_ revealed that despite an elevating DH value during the time-course of hydrolysis, the two hydrolysates represented a different pattern concerning IC_50_ values. The alcalase hydrolysate IC_50_ value showed a downward trend whereas flavourzyme hydrolysate IC_50_ value exhibited mixed pattern trend with two phases: an increase in DH value up to 20% while IC_50_ decreased, but by further increase in DH value again IC_50_ values increased. Alcalase hydrolysate showed an almost steady decrease in IC_50_ values with the progression of hydrolysis. Balti et al. (2010) reported a correlation coefficient of 0.83 between DH and ACE inhibitory activity of cuttlefish muscle hydrolysate prepared using several proteases [32]. Since inhibitory effect of a peptide is affected by the type of amino acids present in its N- and C-terminal sequence which totally depend on the original primary structure and the choice of enzyme then DH and IC_50_ or inhibitory effect might not be correlated in certain cases. 

### 4.5. Hydrolysis-Derived Peptides Molecular Weight Distribution

**Figure 4:**
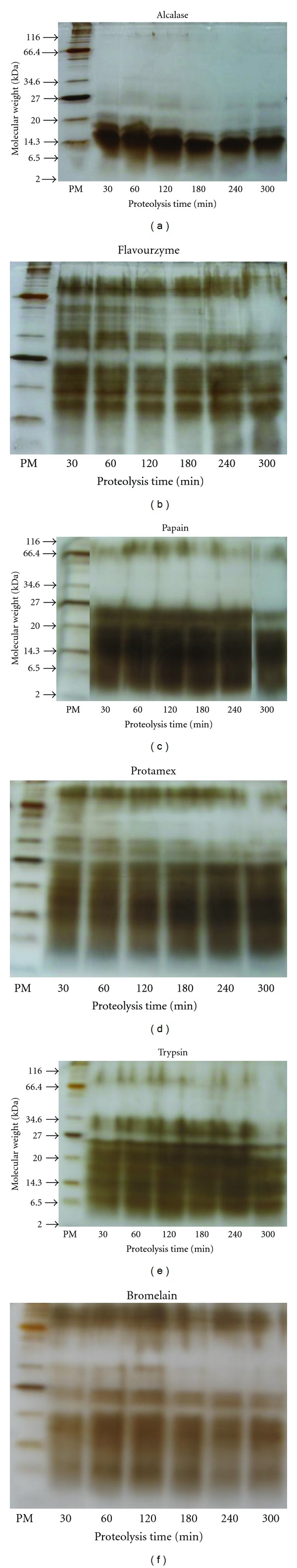
SDS-PAGE of *S. horrens* hydrolysates at 30, 60, 120, 180, 240, and 300 min performed using 15% resolving gel. Each well consists of 3.36 *μ*g protein. The lane indicated as PM was the protein molecular marker.

Since the size of the generated peptides is crucial on the ACE inhibitory effect as previously reported [33, 34], the peptides molecular weight distribution of *S. horrens* hydrolysaes during the proteolysis time was investigated using SDS-PAGE.  Figure 4 revealed that alcalase was found to be the most efficient enzyme towards degrading proteins of *S. horrens* due to the cleavage of nearly all proteins in the high molecular weight region and generation of polypeptides below 20 kDa. This result is in line with the DH and another finding of this study which indicates that alcalase exhibits the lowest IC_50_ value (0.4 mg/mL) compared to the other enzymes. Collagen has been found to be the major protein compound of sea cucumber consisting of *α* chain with the molecular weight of 100 kDa [44, 45]. The absence of high molecular bands above 100 kDa indicates that alcalase is capable of degrading collagen to smaller molecules with molecular weight below 27 kDa. Production of such low molecular weight peptides occurred in this investigation even within the first 30 min of enzyme hydrolysis by alcalase. This finding supports a previous research on alcalase hydrolysate of Sardinella by-product which was reported to generate peptides below 20 kDa with DH value of 10.16% [46].

 On the other hand, papain and trypsin were almost effective towards degrading most of the large protein components most probably including collagen since from the first sample (taken at 30 min of proteolysis) large protein bands (116 kDa) nearly disappeared. The protein range of hydrolysate produced by alcalase, papain and trypsine was below 20, 27, and 34 kDa, respectively. Such lower molecular weight peptides generated by alcalase indicates that *S. horrens* protein could possess the most cutting sites suitable for alcalase in comparison with papain and trypsin enzymes. 

The electrophoretic patterns of the other enzyme hydrolysates were found to be entirely dissimilar and other hydrolysates mostly contained peptides with molecular weight in the range between 4 and 116 kDa. As for hydrolysates prepared by flavourzyme and protamex, protein bands between 34 to 66 were disappeared before 300 min of hydrolysis. In the case of bromelain, protamex, and flavourzyme hydrolysates, the protein bands around 34 kDa were almost hydrolyzed within 5 h. The intensity of the protein bands below 14 kDa, in the case of flavourzyme, increased as the hydrolysis time reached to 300 min. Papain and trypsin were more effective towards degrading most of the larger proteins compared to flavourzyme, protamex, and bromelain. Interestingly, the most marked reduction in the molecular weight of peptide was found in alcalase hydrolysate whose protein intensity was quite high (6 to 20 kDa). Our results were in agreement with those of some previous studies conducted on different substrates, that is, Atlantic cod viscera [47] and red salmon head [48], which revealed high efficiency of alcalase for protein cleavage leading to production of small peptides. 

### 4.6. Amino Acid Composition

 The contents of different amino acids of untreated *S. horrens* and enzyme-treated *S. horrens* (with alcalase, flavourzyme, trypsin, papain, protamex, and bromelain) hydrolysates, calculated on dry weight basis, are given in Table 1. The amino acid profile of untreated sea cucumber showed that glycine dominated among others with content of 66.2 mg/g. This is in agreement with the previous results for *Stichopus japonicas *[44]. Glutamic acid was the second most abundant component (53.8 mg/g) followed by alanine and proline with contribution of 52.5 and 48.2 mg/g, respectively. These results were also consistent with the published work on *H*. *scabra*,* H. nobilis*, *H. impatiens*,* H. multipilula*,* Actinopyga echinites*, and* Thelenota ananas* [25]. A similar trend of amino acid profile was consistently followed by alcalase, trypsin and flavourzyme hydrolysates but varied for protamex, papain, and bromelain hydrolysates. This can be related to the different specificity of each enzyme affecting the sequence of amino acid within peptides solublized by the enzyme action. A significant difference only in terms of tyrosine, and lysine existed in hydrolysates of alcalase, and trypsin. However, flavourzyme hydrolysate gave much higher values for all amino acids compared to those of alcalase or trypsin. When compared with untreated sea cucumber, the amino acid composition of protamex hydrolysate revealed the presence of higher concentration of aspartic acid and thereonine whereas amounts of other amino acids were quite low.

As mentioned before, it is known that hydrophobic and positively charged amino acids existing in peptide C-terminal contribute to the peptide inhibitory properties. Flavourzyme hydrolysate exhibited the highest hydrophobic content followed by alcalase and trypsin hydrolysate. In case of positively charged amino acids (Arg, Lys), almost the same pattern happened; however, it is not comparable with those of IC_50_ values. Alcalase hydrolysate possessed the lowest IC_50_ value followed by flavourzyme, trypsin, and bromelain hydrolysate suggesting that position of those amino acids in the peptide sequence is more crucial than their total amounts.

## 5. Conclusion

This study revealed that enzyme hydrolysates of *S. horrens* have good potential for production of ACE inhibitory peptides and also offered high amounts of valuable amino acids. Of the different proteases tested, alcalase was found to be the most efficient for production of hydrolysate with the best ACE inhibitory activity (IC_50_ value of 0.41 mg/mL). Therefore the belief of sea cucumber having antihypertensive effect in the folk medicine is supported *in vitro*. Thus, *S. horrens* protease hydrolysates could be employed as a potential source of functional ACE inhibitory peptides suggesting their uses for regulating normal blood pressure in hypertensive humans, in addition to obtaining other physiological benefits. 

## Figures and Tables

**Table 1:** Amino acid composition (mg/g dry weight) of *S. horrens* hydrolysates after 5 h hydrolysis using different proteases^a^.

Amino acid	Sea cucumber	Alcalase	Flavourzyme	Trypsin	Papain	Bromelain	Protamex
Asp	22.87 ± 1.00^c^	32.52 ± 3.29^ab^	36.35 ± 4.76^a^	30.23 ± 0.14^b^	10.76 ± 0.02^e^	17.84 ± 0.57^d^	23.60 ± 0.15^c^
Glu	53.85 ± 0.72^b^	56.93 ± 0.00^b^	76.97 ± 4.86^a^	56.34 ± 0.00^b^	24.92 ± 0.02^e^	35.13 ± 1.01^d^	47.36 ± 0.00^c^
Ser	16.00 ± 1.36^b^	15.32 ± 0.65^bc^	20.04 ± 0.20^a^	15.65 ± 0.21^b^	8.13 ± 0.01^e^	11.72 ± 0.46^d^	13.92 ± 0.06^c^
Gly	66.21 ± 1.37^b^	61.57 ± 1.00^c^	88.64 ± 2.58^a^	61.18 ± 0.42^c^	20.78 ± 0.26^e^	21.62 ± 0.93^e^	54.76 ± 0.53^d^
Arg	35.98 ± 0.49^b^	32.98 ± 0.57^c^	40.02 ± 0.72^a^	33.33 ± 0.14^c^	11.42 ± 0.19^f^	14.95 ± 0.00^e^	23.89 ± 0.12^d^
Thr	10.36 ± 0.19^d^	9.62 ± 0.15^d^	23.64 ± 0.54^a^	9.74 ± 0.42^d^	9.58 ± 0.06^d^	13.62 ± 0.75^c^	15.96 ± 0.61^b^
Ala	52.59 ± 0.13^a^	49.42 ± 0.54^b^	41.16 ± 0.85^c^	49.15 ± 0.21^ b^	9.48 ± 0.18^f^	12.40 ± 0.64^e^	24.04 ± 0.60^d^
Pro	48.27 ± 0.13^a^	44.89 ± 0.14^b^	40.09 ± 0.94^c^	43.45 ± 0.35^b^	11.12 ± 0.16^f^	13.44 ± 0.63^e^	25.40 ± 0.78^d^
Tyr	9.29 ± 0.35^b^	8.14 ± 0.02^c^	10.34 ± 0.20^a^	7.59 ± 0.02^d^	4.51 ± 0.02^f^	6.87 ± 0.05^e^	7.24 ± 0.18^de^
Val	16.81 ± 0.90^a^	14.01 ± 0.16^b^	16.92 ± 0.10^a^	13.78 ± 0.04^bc^	7.53 ± 0.01^d^	12.24 ± 0.38^bc^	12.00 ± 0.08^c^
Ile	17.21 ± 0.18^a^	15.13 ± 0.11^b^	16.95 ± 0.17^a^	14.66 ± 0.07^b^	7.98 ± 0.10^d^	12.95 ± 0.59^c^	12.96 ± 0.21^c^
Leu	15.53 ± 0.22^b^	13.96 ± 0.16^c^	16.70 ± 0.38^a^	13.82 ± 0.01^c^	7.06 ± 0.10^f^	12.24 ± 0.50^d^	10.75 ± 0.13^e^
Phe	7.22 ± 0.14^b^	6.44 ± 0.03^c^	7.83 ± 0.52^a^	6.15 ± 0.02^c^	4.23 ± 0.01^d^	7.47 ± 0.40^ab^	6.01 ± 0.07^c^
Lys	11.48 ± 0.04^b^	10.01 ± 0.10^d^	13.48 ± 0.12^a^	10.75 ± 0.02^c^	7.52 ± 0.06^f^	11.60 ± 0.56^b^	9.32 ± 0.06^a^
Hydrophobic AA	281.47	258.51	278.78	253.28	83.86	112.72	153.20
Hydrophilic AA	154.66	160.91	215.30	159.87	74.19	107.75	100.87
positively charged AA	47.47	43.00	53.51	44.09	18.95	26.56	33.21

^a^Data are means ± SD of triplicate determinations. Means with different superscript letters within the same row indicate significant differences among hydrolysates (*P* ≤ 0.05).
